# Influence of Cation Vacancies on Li Conductivity of La_1/2_Li_1/2–2*x*_Sr_*x*_TiO_3_ Perovskites (0 < *x* ≤ 0.25): The Role of Nominal and Effective Vacancies

**DOI:** 10.1021/acsaem.2c03519

**Published:** 2023-03-02

**Authors:** Wilmer Bucheli, Ricardo Jiménez, Jesús Sanz, Maria Eugenia Sotomayor, Alejandro Varez

**Affiliations:** † Departamento de Energía. Instituto Ciencia de Materiales (ICMM-CSIC), 28049 Madrid, Spain; ‡ Departamento de Ciencia e Ingeniería de Materiales e Ingeniería Química, IAAB, Universidad Carlos III de Madrid, Av. Universidad 30, 28911 Leganes, Spain

**Keywords:** perovskites, strontium doping, ^7^Li MAS-NMR spectroscopy, lithium mobility, solid electrolytes, ionic conductors

## Abstract

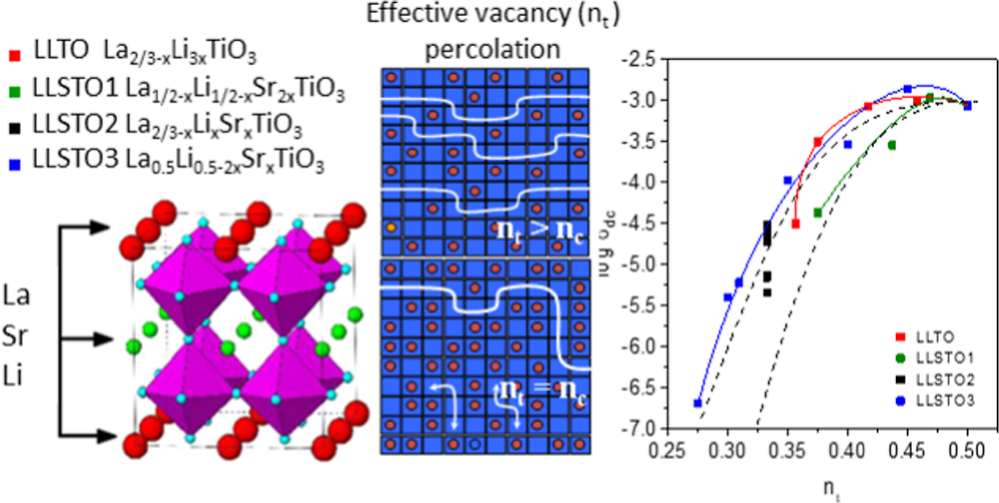

The Li_1/2–2*x*_Sr_*x*_La_1/2_TiO_3_ series (0 ≤ *x* ≤ 0.25) is investigated with X-ray diffraction, nuclear magnetic resonance, and impedance spectroscopy techniques. The substitution of two Li^+^ by one Sr^2+^ in Li_1/2_La_1/2_TiO_3_ perovskite generates cation vacancies that, when ordered in alternating planes along the *c*-axis, confer a two-dimensional character to Li mobility. In previous works, it was shown that Li^+^ ions partially occupy the center of the six faces of the cubic perovskite, resulting in the associated A-sites to participate like a vacancy in the definition of the percolation vacancy threshold. The results obtained in the Li_1/2–2*x*_Sr_*x*_La_1/2_TiO_3_ series are compared with those obtained in the Li_3*x*_La_2/3–*x*_TiO_3_ series, and other Sr-doped solid solutions (Li_1/2–*x*_Sr_2*x*_La_1/2–*x*_TiO_3_ and Li_*x*_Sr_*x*_La_2/3–*x*_TiO_3_), to highlight the importance of the effective vacancies with respect to the nominal ones in conductivity. The analysis of four series, belonging to the ternary SrTiO_3_–La_2/3_TiO_3_–Li_2_TiO_3_ phase diagram, permits a better understanding of the ionic conduction mechanism in perovskites. The results show that the vacancy percolation model is more adequate to explain Li conductivity than the conventional hopping probability model. In the analyzed series, Li conductivity is maximum when a small amount of Sr is incorporated into the pseudo-cubic La_1/2_Li_1/2_TiO_3_ end member, while it decreases as the amount of strontium increases.

## Introduction

The Li_3*x*_La_2/3–*x*_TiO_3_ (LLTO) perovskite series, with 0.03 ≤ *x* ≤ 0.16, has attracted much interest because of its ionic conductivity as high as 10^–3^ S cm^–1^ at room temperature and its potential application as an electrolyte in all solid state batteries.^1−3^ In particular, structural features induced by Li stoichiometry, vacancy distribution, and thermal treatments are of particular relevance.^4^ So, in La-rich LLTO perovskites prepared by slow cooling from 1573 K, a doubled *a*_p_ × *a*_p_ × 2*a*_p_ unit cell (*a*_p_ is the lattice constant of a simple cubic perovskite) was detected, where La and the vacancy are ordered in alternating planes. The structural refinement of X-ray diffraction (XRD) patterns of a La-rich sample showed a doubled orthorhombic perovskite (S.G.: *Pmmm*) with a unit cell of *a*_p_ × *a*_p_ × 2*a*_p_, where Ti cations shift from the center of octahedra toward vacancy-rich planes, producing a clear differentiation of two Ti–O distances along the *c*-axis in octahedra.^5^ Most studies of perovskite were performed with tetragonal^6−8^ or orthorhombic^9^ diagonal √2*a*_p_ × √2*a*_p_ × 2*a*_p_ phases, where cation ordering increased progressively the *c*/2*a* ratio as La content increased. In La-rich perovskites, an orthorhombic superstructure (S.G.: *Cmmm*) 2*a*_p_ × 2*a*_p_ × 2*a*_p_ was detected by neutron diffraction that described properly the octahedral tilting.^10,11^ When LLTO perovskites were quenched from 1573 K into liquid nitrogen, a disordered rhombohedral √2*a*_p_ × √2*a*_p_ × 2√3*a*_p_ cell (S.G.: *R*3̅*c*) was detected.^12^

In the quenched Li_1/2_La_1/2_TiO_3_ perovskite, the strong difference between La^3+^ and Li^+^ charges should favor their alternation along three crystallographic axes, but it has not been observed. This is probably related to the unusual location of Li (unit-cell faces of the perovskite), as demonstrated by ND experiments.^12^ It was finally observed that the number of Li that occupies unit-cell faces of primitive perovskite remains slightly below nominal values, suggesting the presence of some Li vacancies. High conductivity values measured in this perovskite suggest that cation disordering is a key factor to explain the high mobility of lithium in Li-rich LLTO samples.

In LLTO perovskites, there are important features that affect ionic conductivity: (i) the presence of nominal vacancy (□_A_) created by partial aliovalent substitution (i.e., La^3+^ by M^2+^ or M^+^); (ii) the unusual location of Li at square unit-cell faces of the perovskite favoring the partial occupation of six-equivalent sites by Li^+^ ions;^12^ (iii) A-sites associated with lithium, which behaves as a vacancy for conductivity, causing the so-called “effective” vacancy, *n*_*t*_ = [Li] + □_A_, to play an important role in transport properties;^13,14^ and (iv) the La-□_A_ ordering in alternate planes along the *c*-axis, which favors the two-dimensional motion of lithium in doubled perovskites.^15^

The substitution of Li^+^ by Na^+^ ions, in (Li_1–*y*_Na_*y*_)_3*x*_La_2/3–*x*_TiO_3_ (*x* = 0.06 and 0.167) samples, reduced the number of “effective” vacancy, decreasing Li conductivity dramatically when *n*_t_ approaches the vacancy percolation thresholds (*n*_p_) of perovskites with 2D (*n*_p_ ≈ 0.54) or 3D (*n*_p_ ≈ 0.31) mobility.^13−15^ In Li_3*x*_La_2/3–*x*_TiO_3_ perovskites, the amount of “effective” vacancies can be modified by substituting La^3+^ by less-charged Sr^2+^ and/or Li^+^ cations.

**Figure 1:**
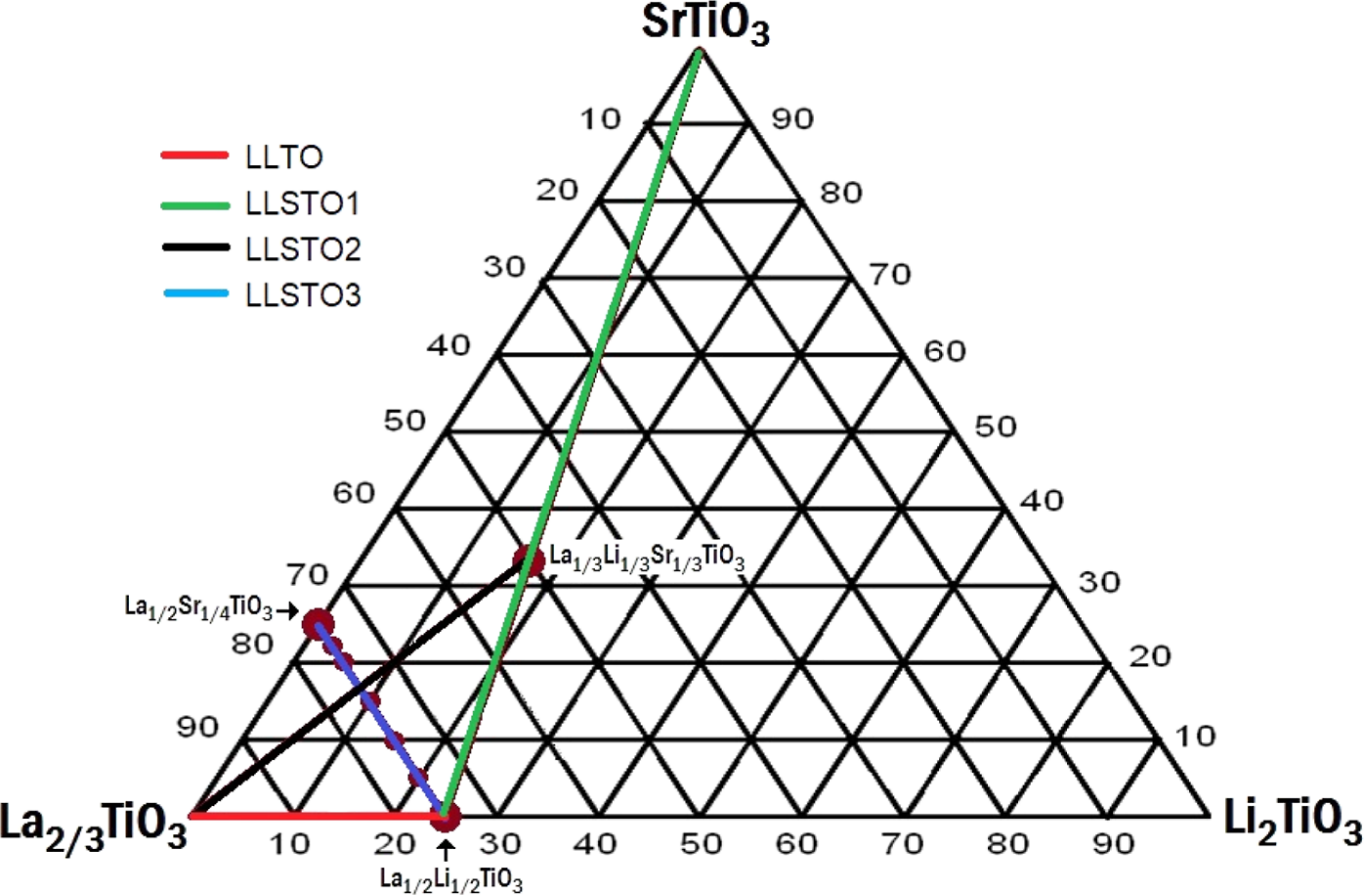
Ternary SrTiO_3_–La_2/3_TiO_3_–Li_2_TiO_3_ phase diagram, where different Sr^2+^ substitution schemes are visualized. The blue line stands for the series investigated in this work, and the red, green, and black lines were analyzed in refs (16)(17), and (18).

In previous works, Inaguma et al.^16^ showed that the incorporation of Sr^2+^ in the Li_3*x*_La_2/3–*x*_TiO_3_ (LLTO) series increased the unit cell volume and stabilized the cubic phases, achieving one of the best reported conductivity values for perovskites. In our group, two series were previously analyzed: the LLSTO1:Li_1/2–*x*_Sr_2*x*_La_1/2–*x*_TiO_3_ series^17^ and LLSTO2:Li_*x*_Sr_*x*_La_2/3–*x*_TiO_3_ series,^18^ denoted by green and black lines in Figure 1. In the first series, the symmetry was always cubic, but in the second one, the structure displayed a progressive transition from orthorhombic to tetragonal and finally cubic symmetry. In both analyzed series, the Li_1/3_Sr_1/3_La_1/3_TiO_3_ sample was prepared (the intersection point of green and black lines), where three cations were randomly distributed in one crystallographic site and conductivity displayed a three-dimensional character.

In the present work, the LLSTO3:La_1/2_Li_1/2–2*x*_Sr_*x*_TiO_3_ series (blue in Figure 1) is analyzed, where the substitution of two Li^+^ by Sr^2+^ increases the number of nominal vacancies up to □_A_ = 1/4 in the Sr_1/4_La_1/2_TiO_3_ end member. The distribution of cations and vacancies is deduced from XRD structure refinements. The Li mobility is analyzed with ^7^Li NMR and impedance spectroscopy (IS). In the Discussion section, the transport properties of this series are compared with those of LLSTO1:Li_1/2–*x*_Sr_2*x*_La_1/2–*x*_TiO_3_^17^ and LLSTO2:Li_*x*_Sr_*x*_La_2/3–*x*_TiO_3_ series,^18^ where *n*_t_ decreases or remains constant, respectively. In particular, the role played by the amount and distribution of vacancies is discussed to better deduce factors that govern ionic conductivity in perovskites.

## Experimental Section

Li_1/2–2*x*_Sr_*x*_La_1/2_TiO_3_ perovskites (0 < *x* ≤ 0.25) were prepared by solid state reaction from stoichiometric amounts of Li_2_CO_3_, SrCO_3_, TiO_2_, and La_2_O_3_ reagents following refs (18) and (19). Before weighing, reagents were heated at 300 °C to eliminate adsorbed water. In the case of La_2_O_3_, powders were dried and de-carbonated at 800 °C. These reagents were ground together in an agate mortar and heated at 800 °C for 12 h. The reground products were cold-pressed at 150 MPa and heated at 1150 °C for 12 h. Finally, powders were uniaxially pressed and heated at 1 °C/min from 300 to 1250–1400 °C for 6 h. The optimum sintering temperature was reduced from 1350 to 1250 °C as Sr content increased, avoiding the adhesion of samples to the alumina crucible. To avoid lithium losses, the compacts were covered with powders of the same composition.

The XRD technique was used to follow the assessment of formed phases. The XRD patterns analysis was performed using the Fullprof program. With the LeBail technique, the zero pattern, the line base, and the line width of peaks were determined. In structural refinements of doubled perovskites (S.G.: *P*4/*mmm*, no. 123), unit-cell parameters were first determined, and then, positions of atoms, site occupancy, and atoms’ thermal factors were deduced. The sample composition was fixed during refinements, but in the last runs, it was varied. To analyze the cation distribution, differences in La, Sr, and Li contents of contiguous *c*-planes were investigated.

^7^Li (*I* = 3/2) MAS-NMR experiments were performed in an AVANCE 400 (Bruker) spectrometer. ^7^Li NMR spectra were recorded at 155.45 MHz in the presence of the magnetic field *B*_0_ = 9.4 T. The NMR detection was produced after π/2 pulse irradiation, on samples spun at 20 kHz around an axis inclined at 54°44′ with respect to the external magnetic field (MAS-NMR technique). The number of accumulations was 200, and the time used between accumulations was 10 s. ^7^Li chemical shift values were referred to a 1 M LiCl solution.

The temperature dependence of conductivity was investigated by IS in the frequency range 20 Hz–1 MHz, using an Agilent E4192A apparatus. Sintered pellets were 9 mm in diameter and around 1 mm in thickness. Electrical contacts (gold paste) were deposited on the parallel surfaces of pellets and then heated at 1125 K. Electrical measurements were performed under a nitrogen atmosphere, with a 4TP four-terminal pair configuration, between 77 and 550 K (10 K intervals) in a JANIS VPF 750 cryostat. For low-frequency measurements (1 mHz–10 kHz), a Zaner IM6ex apparatus was used. In the temperature range analyzed, the electronic contribution remained below 0.05% of the total conductivity.

## Results

### XRD Study

**Figure 2:**
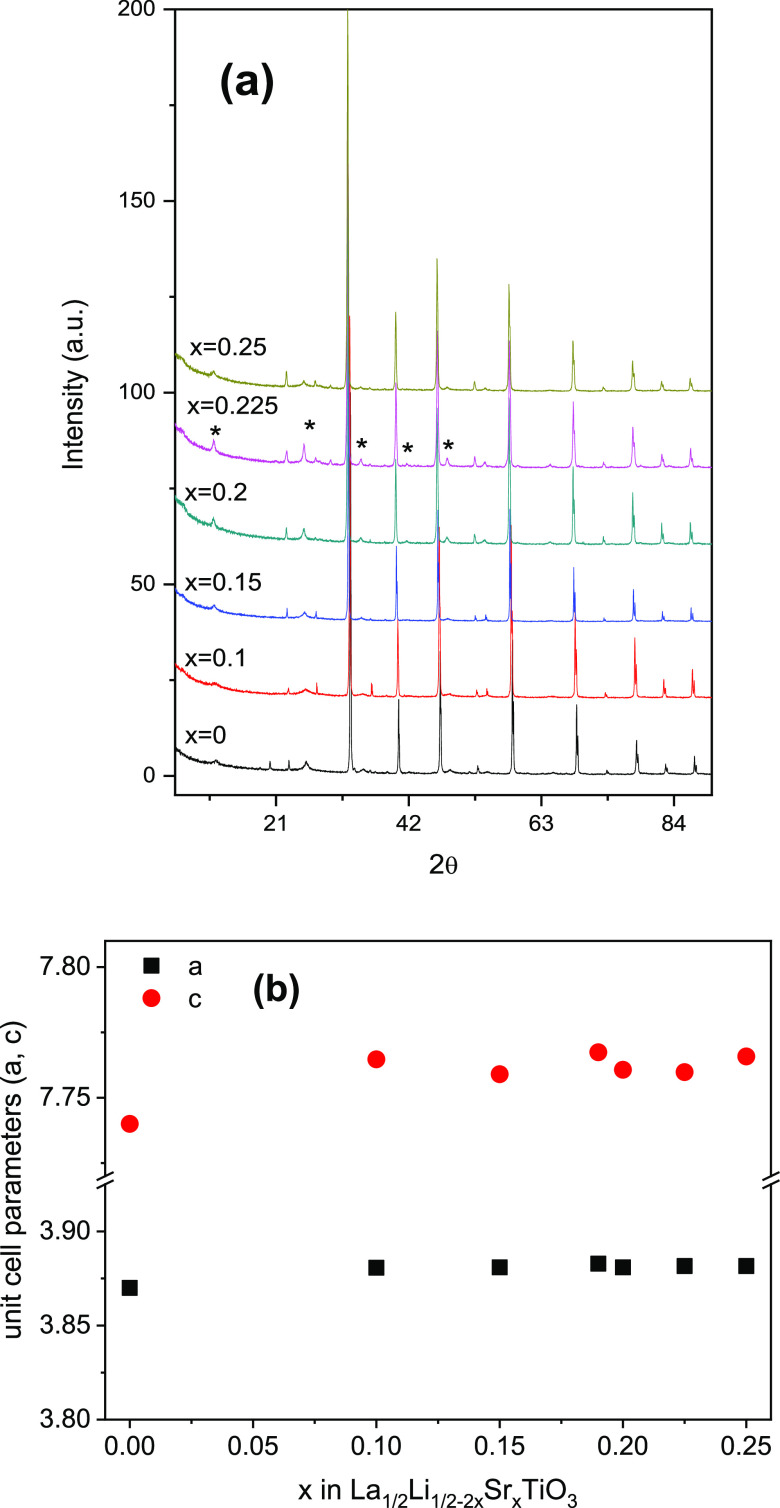
(a) XRD patterns and (b) unit cell parameters of the La_1/2_Li_1/2–2*x*_Sr_*x*_TiO_3_ (LLSTO3) series.

The XRD patterns of the perovskite LLSTO3:La_1/2_Li_1/2–2*x*_Sr_*x*_TiO_3_ series (0 < *x* ≤ 0.25) are depicted in Figure 2a. For the end member Li_1/2_La_1/2_TiO_3_, the XRD patterns can be indexed with a cubic single perovskite (*a*_p_ × *a*_p_ × *a*_p_), but at increasing Sr contents, the main peaks of a doubled cell (*a*_p_ × *a*_p_ × 2*a*_p_), labeled with asterisks in XRD patterns, were detected. These peaks are rather broadened compared to those of the cubic perovskite (*a*_p_ × *a*_p_ × *a*_p_), which is normally associated with the formation of micro- or nanodomains. These nanodomains have been visualized in other Sr-doped samples.^19^

In the second stage, structural refinements were performed with the Rietveld technique. In this analysis, a double tetragonal perovskite, *a*_p_ × *a*_p_ × 2*a*_p_ (S.G.: *P*4/*mmm*, no. 123), was adopted for comparative purposes. In Table S1 of the Supporting Information, the structural model used is detailed. Along the series, the unit cell parameters increased with Sr, in agreement with the ionic radii, but remained almost constant for the highest contents (Figure 2b). In refinements, atoms positions, site occupancy, and thermal factors were deduced. The refinement results, including agreement factors and parameters deduced for different samples, are given in Table S2. In the Li-rich member Li_1/2_La_1/2_TiO_3_ (*x* = 0), six Ti–O distances are equal in octahedra, but in Sr-rich contents, the deduced distances differ as a consequence of the vacancy ordering in alternating planes. In these samples, Ti–O distances are close to 1.94 Å in *ab*-planes, but the distances along the *c*-axis increase and decrease, respectively. Considering that the average A–O distances are defined by lanthanum (La–O ∼ 2.5 Å), the Li ions are forced to shift toward square windows that connect contiguous A-sites, to reduce Li–O distances to ∼2.0 Å values, as demonstrated by ND experiments.^12^

**Figure 3:**
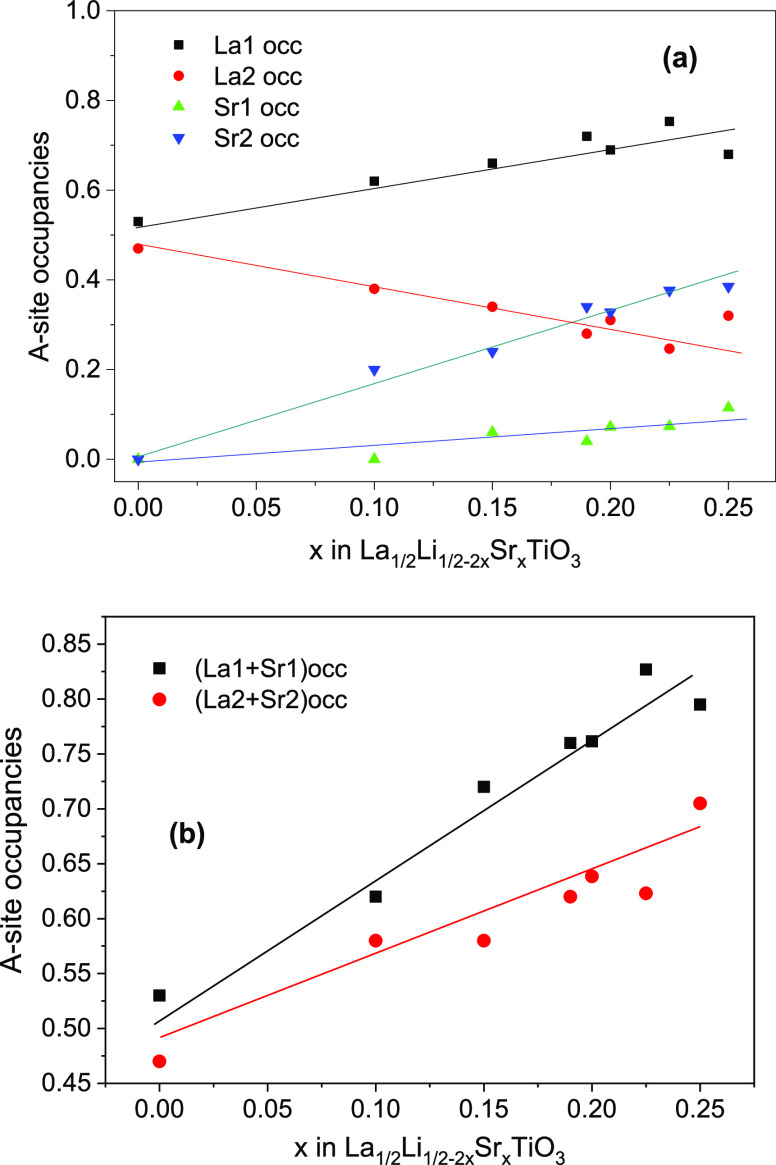
(a) La and Sr occupancy of two sites; (b) occupancy of alternating planes along the *c*-axis in the La_1/2_Li_1/2–2*x*_Sr_*x*_TiO_3_ series.

An analysis of the site occupancy shows that adjacent planes are equally occupied by La in the Li-rich end member (La_1/2_Li_1/2_TiO_3_). This is compatible with a cubic unit cell, but occupancies differ with increasing Sr (vacancy) content, as a consequence of the La, Sr, and vacancy ordering (Figure 3). As a result of cation ordering, a certain preference of La for 1a site and Sr for 1b site was detected (Figure 3a and Table S2). As Sr increases, the occupation of the two sites become different, favoring a 2D mobility of lithium (Figure 3b).

### NMR Results

**Figure 4:**
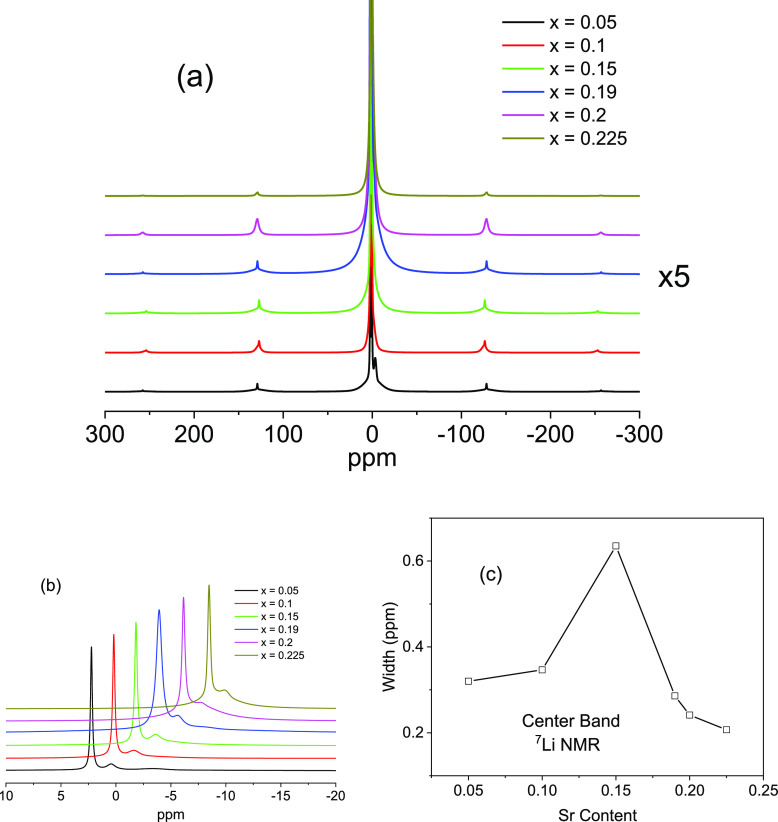
(a) ^7^Li MAS-NMR spectra of La_1/2_Li_1/2–2*x*_Sr_*x*_TiO_3_ samples. The spinning sideband patterns of samples display very low intensity (×5 magnification used). (b) Spectra are slightly shifted by a constant value for a better visualization of central components. (c) Plot of linewidths of components against the Li content of samples.

The ^7^Li MAS-NMR spectra of Li ion conductors are formed by the central band and equally spaced side bands, produced by the sample rotation, at both sides of the spectrum. In the case of the La_1/2_Li_1/2–2*x*_ Sr_*x*_TiO_3_ series, the central component at ∼2 ppm was preponderant, but spinning side band patterns displayed lower intensity, making necessary a ×5 magnification for their visualization (Figure 4a).

In the Li-rich sample (*x* = 0.05), spinning side bands display low intensity because Li mobility cancels dipolar Li–Li interactions (Figure 4a). When Li content decreases, dipolar interactions decrease, making visible quadruple interactions. However, the quadrupole patterns always remain small because local symmetry of Li sites is nearly cubic. In Sr-rich samples, quadruple interactions decrease because of residual mobility of lithium and pseudo-cubic local symmetry of Li sites.

In agreement with these ideas, the line width of the central component was minimum in Li-rich samples, indicating that mobility was important (Figure 4b). When Sr content increases, the line width increases up to *x* = 0.15 (Figure 4c). This observation has been ascribed to the transition from a cubic to tetragonal symmetry, which enlarges the line width of the signal when both phases coexist. When samples are mostly tetragonal, vacancies become ordered and the line width decreases (Figure 4c).

In samples analyzed here, a second narrow component was detected near 0 ppm, which was associated with the exchange of lithium by protons of adsorbed water.^20^ This component was ascribed to the formation of LiOH at the perovskite surface. The surface carbonation of LiOH increases the grain-boundary contribution to the total resistance of the sample. The Li/H exchange is relevant in Li-rich samples but decreases when the Sr content increases (Figure 4b). In Sr-rich samples, a new component at −3.5 ppm was detected that must be ascribed to the formation of Sr-rich secondary phases with lithium.

### Electrical Characterization

**Figure 5:**
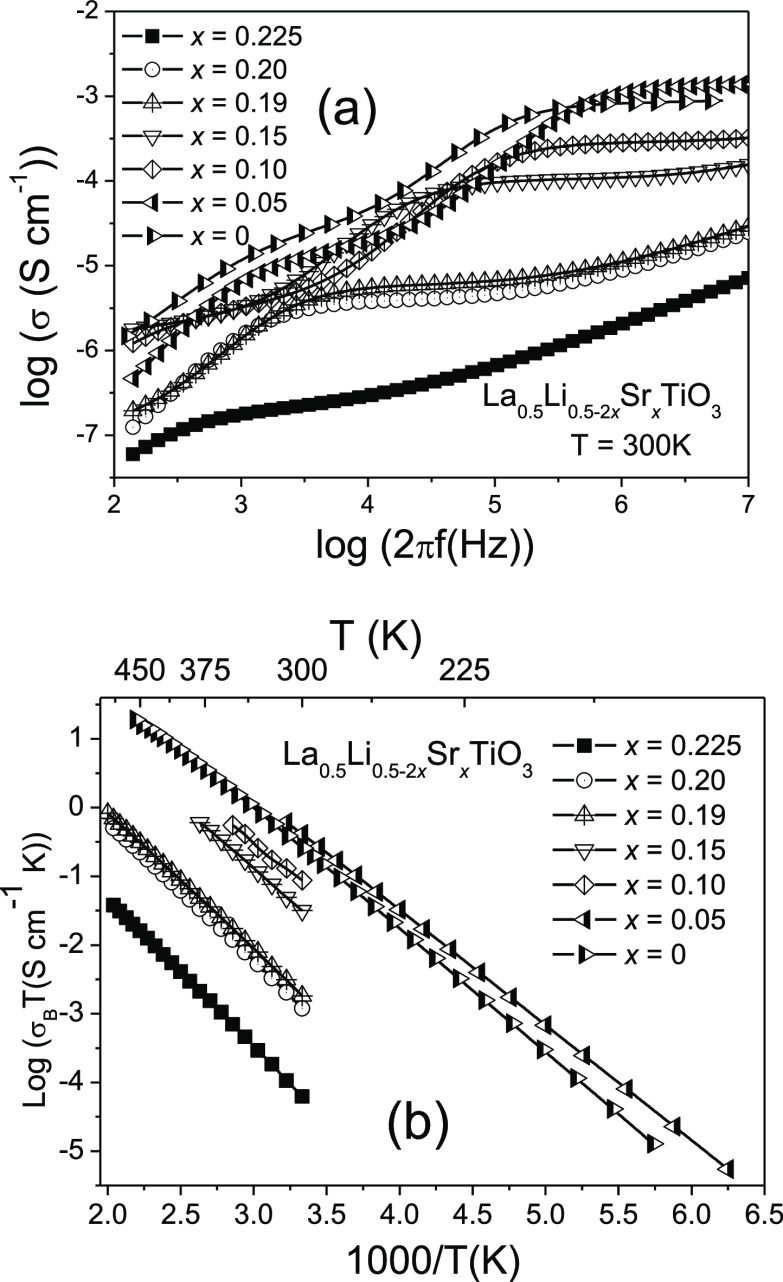
(a) Frequency dependence of conductivity in the La_1/2_Li_1/2–2*x*_Sr_*x*_TiO_3_ series. (b) Plot of dc-conductivity vs inverse of temperature.

In Figure 5a, the frequency (ω) dependence of conductivity at 300 K is given for samples with different *x* values. For *x* = 0.05, bulk (ω ≅ 10^6^–10^7^ s^–1^), grain-boundary (ω ≅ 10^3^–10^4^ s^–1^), and electrode (ω < 10^3^ s^–1^) contributions are resolved in the conductivity plots. For a higher Sr content, the three conductivity contributions are shifted toward lower frequency.

To obtain the “bulk” and “overall” conductivities of the ceramic samples, and following the previously developed methodology, the log(σ) vs log(ω) derivative plot was used.^21^ By this procedure, the minimum values of the log(σ) vs log(ω) derivative plot in the frequency regions where “bulk” and “grain-boundary” conductivity are dominant have been used to obtain the corresponding conductivity values from the log(σ) vs log(ω) plot. This simplified procedure retains a high accuracy on the parameter’s determination.

**Figure 6:**
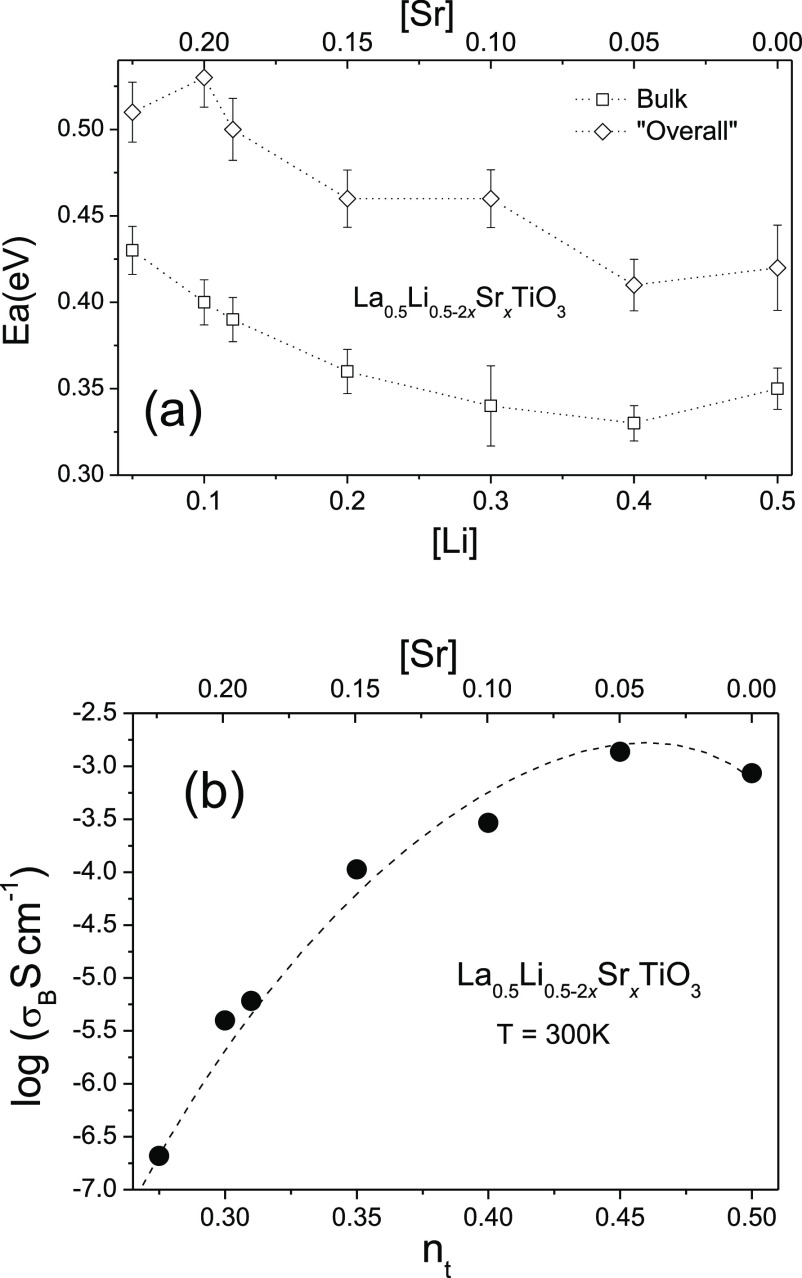
(a) Compositional dependence of activation energy of bulk and overall contributions in La_1/2_Li_1/2–2*x*_Sr_*x*_TiO_3_ samples. (b) Dependence of dc-conductivity on the “effective” vacancy concentration.

The temperature dependence of log(σ*T*) values with the inverse of temperature is shown in Figure 5b. The activation energy values corresponding to the bulk conductivity decrease from 0.43 to 0.32 eV when Li content increases (Sr decreases), increasing slightly in the Li_1/2_La_1/2_TiO_3_ sample (Figure 6a). A similar trend shows the corresponding activation energies associated with the “overall” conductivity, increasing from 0.42 to 0.53 eV, when the Sr content increases (Li content decreases).

When the dc-bulk conductivity of samples is plotted as a function of *n*_t_, the RT conductivity increases with *n*_t_, passing through a maximum (2 × 10^–3^ S/cm) at *n*_t_ = 0.45 (Figure 6b). The subsequent decrease observed in the conductivity is associated with the increases of Sr cations, reducing the *n*_t_ values.

## Discussion

To better understand Li mobility in Sr-doped LLTO perovskites, we have compared the results deduced for the LLSTO3 (La_1/2_Li_1/2–2*x*_Sr_*x*_TiO_3_) series analyzed in this work with those of two series previously reported (LLSTO1: Li_1/2–*x*_Sr_2*x*_ La_1/2–*x*_TiO_3_ and LLSTO2 Li_*x*_Sr_*x*_La_2/3–*x*_TiO_3_) (see Figure 1). For this purpose, we have related structural features deduced by XRD, with NMR and conductivity results.

### Effective Vacancy vs Nominal Vacancy

**Figure 7:**
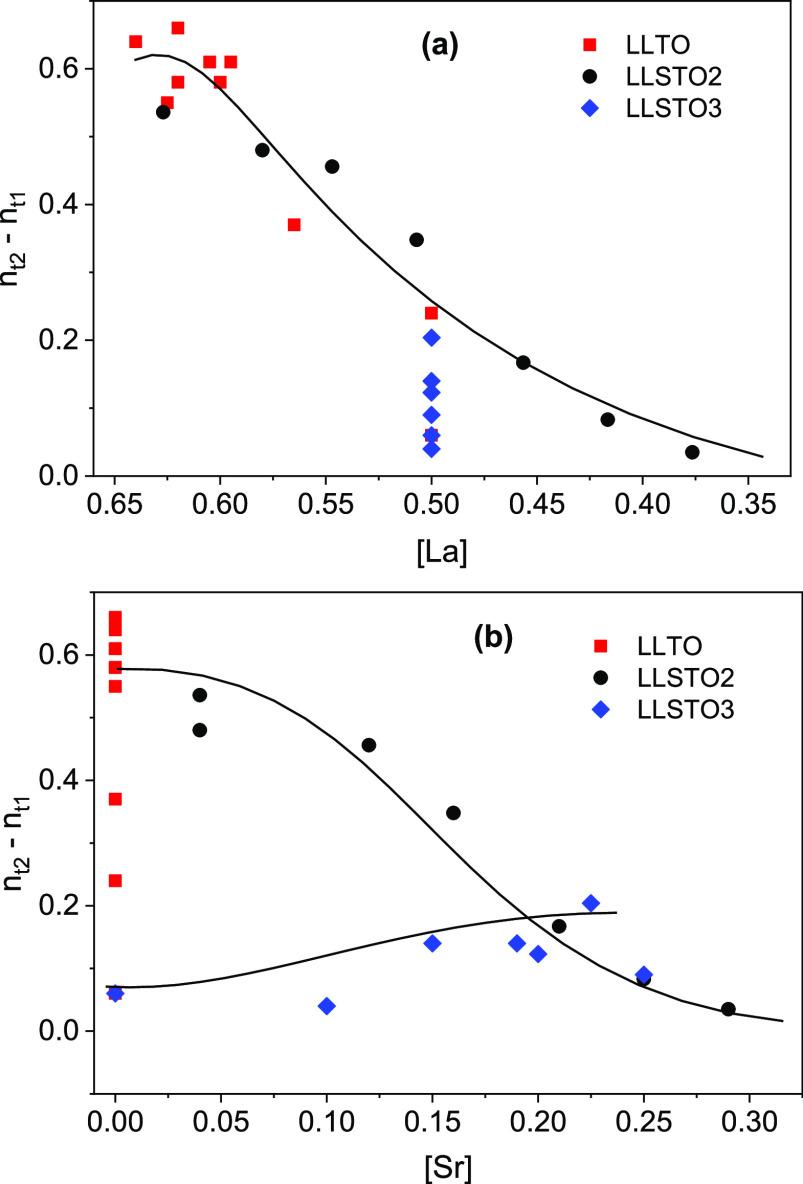
Dependence of nominal vacancy differences of alternating planes on the (a) La and (b) Sr contents of perovskites.

In these perovskites, the nominal vacancies, □_A_, are associated with the aliovalent substitution of La^3+^ by less charged Sr^2+^ or Li^+^ cations in the A-sites. The unusual location of lithium at the center of the six equivalent square faces of the primitive perovskite unit-cell causes the A-sites associated with lithium to behave as a vacancy.^12^ In the case of the common end member, Li_1/2_La_1/2_TiO_3_, of the series LLTO, LLSTO1, and LLSTO3 without Sr, the number of nominal vacancies, □_A_, is zero but exhibits RT ionic conductivity values of approximately 2 × 10^–3^ S/cm (Figure 7). This fact justifies the existence of other vacant positions in the perovskite structure, which participate in the conduction process. The consideration of “effective” vacancy, *n*_t_ = [Li] + □_A_, whose number is higher than those of nominal vacancies, helps to understand the Li conductivity in these perovskites.^13^

To highlight the importance of *n*_t_ on the ionic conductivity of Sr-doped LLTO perovskites, we have considered the three solid solutions of Table 1, which have been strategically selected to analyze the evolution of nominal and effective vacancies throughout the series.

**Table 1:** Chemical Formula, Substitution Scheme, Nominal, and Effective Vacancies in the Analyzed Series

series	chemical formula	substitution scheme	[Li]	nominal vacancies (□_A_)	effective vacancies *n*t = [Li] + □_A_
LLTO	Li_3*x*_La_2/3–*x*_TiO_3_ (0 ≤ *x* ≤ 0.167)	1 La^3+^ ↔ 3 Li^+^	3*x*	1/3 – 2*x*	1/3 + *x*
LLSTO1	Li_1/2–*x*_Sr_2*x*_La_1/2–*x*_TiO_3_ (0 ≤ *x* ≤ 0.5)	1 La^3+^ + 1Li+ ↔ 2 Sr^2+^	1/2 – *x*	0	1/2 – *x*
LLSTO2	Li_*x*_Sr_*x*_La_2/3–*x*_TiO_3_ (0.04 ≤ *x* ≤ 0.33)	1 La^3+^ ↔ 1 Sr^2+^ + 1Li^+^	*x*	1/3 – *x*	1/3
LLSTO3	Li_1/2–2*x*_Sr_*x*_La_1/2_TiO_3_ (0 ≤ *x* ≤ 0.25)	2 Li^+^ ↔ 1 Sr^2+^	1/2 – 2*x*	*x*	1/2 – *x*

**Figure 8:**
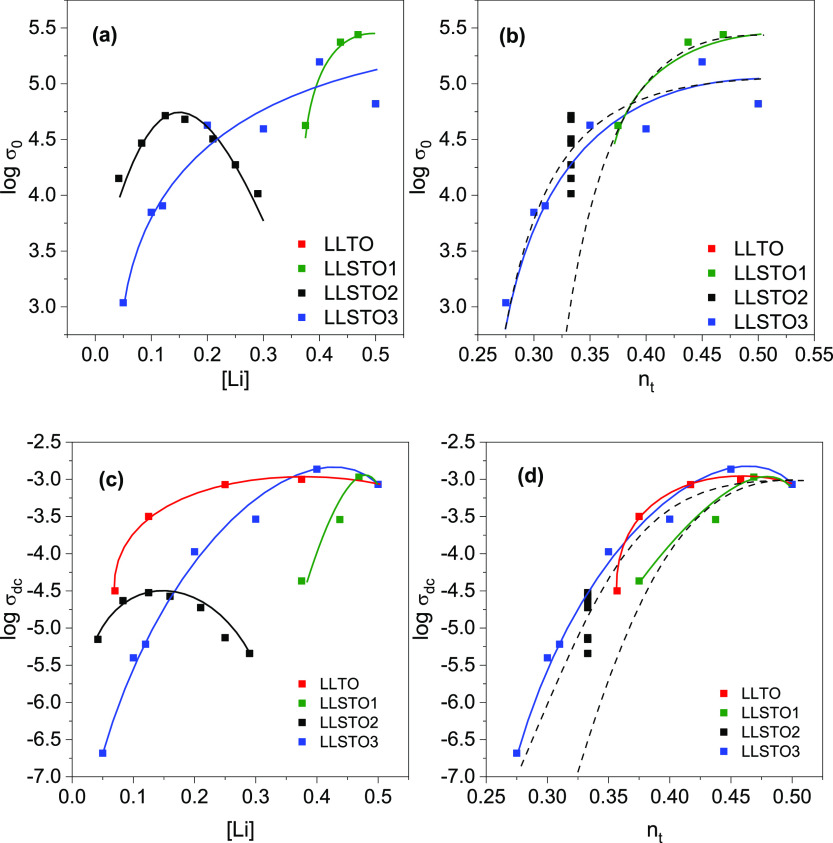
Dependence of (a,b) pre-exponential factors of conductivity and (c,d) dc-conductivity values on Li and *n*_t_ vacancy contents in the SrTiO_3_–La_2/3_TiO_3_–Li_2_TiO_3_ system. Continuous lines are used to guide the eye. Discontinuous lines in (b) and (d) are used to visualize 2D and 3D percolation curves; 2D ∝ (*n*_t_ – 0.27)^2^, 3D ∝ (*n*_t_ – 0.31)^2^.

In the LLSTO1 series, Li_1/2–*x*_Sr_2*x*_La_1/2–*x*_TiO_3_, both La^3+^ and Li^+^ are replaced by two Sr^2+^ cations in the same proportion, causing no nominal vacancies to be produced. The perovskite unit cell deduced by XRD is cubic (*a*_p_ × *a*_p_ × *a*_p_), and La and Sr are randomly distributed on a unique site. The Li conductivity increases slightly with Sr content, achieving a maximum at *x* = 0.05, when the unit cell expanded. Afterward, conductivity decreases by 3 orders of magnitude. This behavior cannot be explained considering nominal vacancies because they remain constant and equal to zero (Figure S1). The variation in *n*_t_ along the series (Figure 8d) explains better conductivity results, giving a percolation threshold, *n*_p_, near that expected for three-dimensional systems (*n*_p_ = 0.31).

In the LLSTO2 series, La_2/3–*x*_Li_*x*_Sr_*x*_TiO_3_, one La^3+^ was substituted by 1 Li^+^ and 1 Sr^2+^, keeping the charge balance but progressively decreasing the nominal vacancies. However, in this case, *n*_t_ remains constant along the series (*n*_t_ = 1/3). The average structure changes from tetragonal to a primitive single cubic as Sr increases. In samples with vacancy ordering (tetragonal phases), TiO_6_ octahedra are distorted, but in cubic phases, octahedra become regular. The evolution of conductivity along the series does not undergo a deep change, displaying a maximum for intermediate compositions, when Li mobility changes from the 2D to 3D regime. The results observed here are similar to those detected in the LLTO series, Li_3*x*_La_2/3–*x*_TiO_3_, where the conductivity only changes slightly when the mobility changes dimensionality. In this series (LLTO), the amount of *n*_t_ always remains above the percolation threshold (*n*_p_) (Figure S1).

In the LLSTO3 series, La_1/2_Li_1/2–2*x*_Sr_*x*_TiO_3_, the number of nominal vacancies increases from 0 to 0.25, but “effective” vacancies decrease with Sr content (*n*_t_ = 1/2 – *x*) from 0.5 to 0.25 (Figure S1). In these samples, the La content remains constant and La is preferentially allocated at 1a sites (S.G.: *P*4/*mmm*, Table S1), while Sr occupies the 1b site. Because of this fact, the occupancy of 1a is higher, favoring the two-dimensional mobility of lithium in alternating planes. In this series, conductivity decreases by several orders of magnitude as the Sr content increases, indicating the onset of 2D percolative processes.

To analyze the cation distribution in perovskites, differences in La, Sr, and *n*_t_ contents of adjacent *ab* planes are investigated. Considering the low sensitivity of XRD to allocate light elements like lithium, the study is centered on the La and Sr site occupation differences. From structural XRD refinements performed in three series, differences on *n*_t_ of alternating planes are estimated with the expression
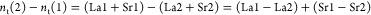
1where “effective” vacancies at structural sites are given by the expression *n*_t_(*S*) = 1 – (La(*S*) + Sr(*S*)) with *S* = 1 or 2.

In the case of the LLTO series, the vacancy ordering is related to that of La, but in the Sr-doped LLSTO series, vacancy ordering is due to La and to a lower degree due to the Sr content (see Figure 7a,b). The LLSTO1 series cannot display La- or Sr-vacancy ordering (not considered in Figure 7). In the LLSTO3 series, vacancy slightly increases with the Sr content (La remains constant). Finally, in the LLSTO2 series, the cation disorder increases as the amount of La decreases. From the above considerations, Li mobility becomes two dimensional when the vacancy becomes ordered. In all cases, the vacancy ordering increases when the amount of La or Sr increases at the expense of the Li content, ordering being more important in La-rich samples.

### Li Conductivity

In general, the long-range dc-conductivity, σ_dc_, can be expressed as
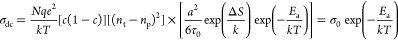
2where *N* and *q* are the charge carrier concentration and charge of lithium, *c* and (1 – *c*) are the Li and vacancy occupations of structural sites, *n*_t_ and *n*_p_ are the amount of “efficient” A-site vacancy and the percolation threshold, *a* is the hopping distance and τ_0_ is the residence time at infinite temperature, and Δ*S* and *E*_a_ are entropy and activation energy for extended Li diffusion motions.

The Li hopping increases when the probability of finding vacancy at the first neighbor site increases. In the presence of a unique site, with *c* and (1 – *c*) denoting Li and vacancy/site occupations, the probability of lithium hopping is given by the expression *c*(1 – *c*). In the analyzed series, the resulting maxima of conductivity should change with lithium and vacancy concentrations.

Depending on the Li and vacancy allocation, at least three different models can be imagined for Li hopping. If Li and vacancy are allocated at A-sites (model 1), Li conductivity is proportional to *c*(1 – *c*) = 3*x*(1/3 – 2*x*) in the LLTO series (Li_3*x*_La_2/3–*x*_TiO_3_), displaying a maximum near [Li] = 0.25, which has not been observed.^14^ The localization of Li at unit cell faces of the primitive perovskite, deduced by ND (model 2), makes Li hopping probability proportional to 3*x*(3 – 3*x*), which should give a maximum in conductivity at [Li] = 1.5, that is, out of the compositional range analyzed.

Both models assume the presence of a well-defined maximum but deviate from experimental results, where: (1) negligible values are detected below a certain composition, (2) a monotonous increment is observed at intermediate compositions, and (3) almost constant values are produced at the highest Li contents. In order to explain these experimental results, a new model (model 3) was proposed where “effective” vacancies (*n*_t_) are considered instead of the nominal A-site vacancy.^13^ In this model, Li conductivity should be controlled by the distribution of vacant A sites, displaying a percolative behavior described by the approximate relation (*n*_t_ – *n*_p_)^2^, where *n*_p_ is the percolation threshold (see eq 2). This model reproduces experimental results in the Li_0.5–*x*_Na_*x*_La_0.5_TiO_3_ and Li_0.2–*x*_Na_*x*_La_0.6_TiO_3_ series.^22^ In 3D systems, *n*_p_ is ∼0.31,^14^ and in 2D systems, it is ∼0.27. In the second series, the alternance of conducting and non-conducting planes makes the mean *n*_p_ value 0.54/2 = 0.27.^18^

In order to analyze the compositional dependence of mobility, the evolution of dc-conductivity with the Li content for the different series is displayed in Figure 8c. In the LLSTO1 and LLSTO3 series, *n*_t_ decreases as the Sr content increases, causing a decrease in the conductivity of several orders of magnitude when the Li content decreases (percolation processes). However, in the LLSTO2 series, where *n*_t_ remains constant, dc-conductivity values are between 10^–5.6^ and 10^–4.5^ S cm^–1^, and conductivity displays a maximum around [Li] ∼ 0.15. When we analyze the evolution of dc-conductivity plots with *n*_t_ (Figure 8d), lower dispersions are found, confirming that *n*_t_ is a more adequate parameter than the Li content to describe conductivity results.^13,15^

From the expression of conductivity, it is also possible to deduce pre-exponential factors σ_0_. The dependence of pre-exponential factors, σ_0_, on the Li content or *n*_t_ is given in Figure 8a,b. The evolution is similar to that of conductivity but somewhat more attenuated, suggesting an important contribution of the vacancy disorder. From this analysis, it can be concluded that the percolation of “effective” vacancy describes better experimental results than the lithium hopping probability.

Depending on the vacancy ordering, different dimensionalities have been observed in the analyzed series. In the LLTO series, the three-dimensional percolation threshold (*n*_p_ = 0.31) is not attained (*n*_t_ > *n*_p_), but in the LLSTO1 series, the 3D percolation process is detected. In the LLTO and LLSTO3 series, the increment of the vacancy ordering (La and Sr content) causes the Li mobility to change progressively from the 3D to 2D regime (see Figure 8d), and conductivity data display 2D percolation processes (average *n*_p_ = 0.27). In LLSTO3 series, although nominal vacancies increase with the Sr content, *n*_t_ decreases, first achieving the 3D (*n*_p_ ∼ 0.31) and then the 2D (*n*_p_ ∼ 0.27) percolation threshold. According to this, the Li conductivity decreases by several orders of magnitude when Sr increases. Similar results are obtained from the analysis of the dimensionality of the samples (Figure 7).

In the Sr-rich end member of the LLSTO3 series, *x* = 0.25, the amount of “effective” vacancies is 0.25, which is below that of the two-dimensional system, *n*_p_ ∼ 0.27. However, the Li mobility is still considerable, suggesting that the percolation threshold is not attained. In structural refinements of these Sr-rich samples, a partial ordering of Sr and La in alternating *ab* planes of perovskites is observed. Based on this fact, it can be assumed that either not all Sr has been incorporated or vacancies are ordered in *ab* planes of the perovskite, precluding the definition of the percolation threshold.

Finally, in LLSTO2 perovskites, where *n*_t_ = 1/3, dc-conductivity should be constant. However, as shown in Figures 7 and 8c, dc-conductivity displays the maximum with the Li content. This fact suggests that, in addition to *n*_*t*_, the jump probability of Li to a free vacancy has some influence on the conductivity, and therefore, the expression *c*(1 – *c*) influences the compositional dependence of conductivity.

An analysis of pre-exponential factors, σ_0_, with Li content or *n*_t_ is given in Figure 8a,b. The evolution is similar but somewhat more attenuated than the conductivity, suggesting an important contribution of vacancy disorder to conductivity.

Following the above considerations, the bulk dc-conductivity must be discussed at two different scales. At short distances, the fast hopping of lithium between six equivalent positions (faces of the single perovskite unit cell) described by *c*(1 – *c*) increases local motion but does not produce necessarily long-range movements.^23,24^ For long distances, the presence of percolated conduction paths along the crystals is required to provide continuity to the lithium migration. In this case, the (*n* – *n*_p_)^2^ expression describes this contribution. Finally, the product *c*(1 – *c*) × (*n* – *n*_p_)^2^ is previously used to reproduce the compositional dependence of conductivity.^13,14^

### Composition Dependence of Conductivity

**Figure 9:**
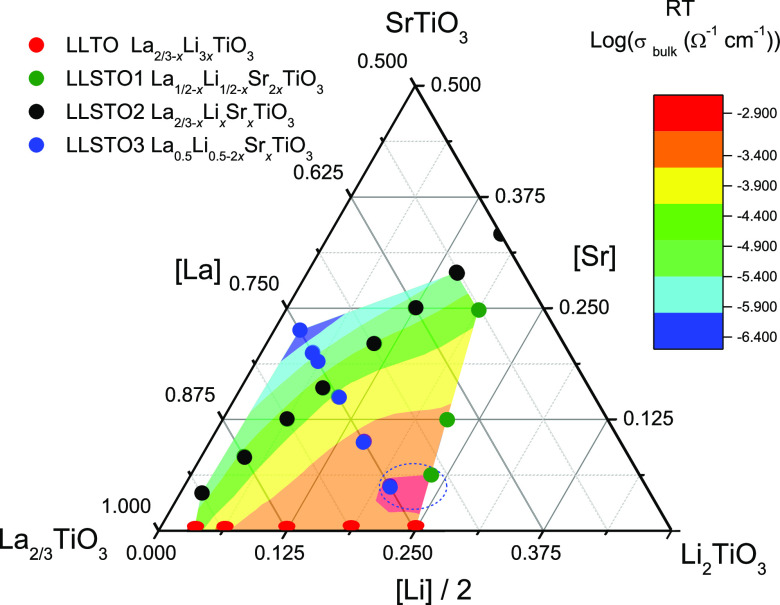
Influence of the sample composition on the conductivity of the investigated series of the SrTiO_3_–La_2/3_TiO_3_–Li_2_TiO_3_ system. Samples with maximum conductivity are displayed near the La_0.5_Li_0.5_TiO_3_ end member (pink region). Samples affected by percolation problems are denoted by blue or green. Finally, secondary Li_2_CO_3_ and SrTiO_3_ phases were detected in Li- and Sr-rich samples. The dashed circle is the region of maximum conductivity in the studied series.

At this point, it is interesting to discuss conductivity values as a function of samples composition (Figure 9). The analysis of dc-conductivity showed that Li-rich samples display higher conductivity than La-rich samples, confirming that three-dimensional disordered conductors, with compositions near Li_1/2_La_1/2_TiO_3_, display the highest conductivity values. In the case of the LLSTO3 series, the maximum RT conductivity is detected in cubic (*x* ∼ 0.02) samples, 4 × 10^–3^ S cm^–1^. The stabilization of the cubic phase, however, does not improve necessarily the Li conductivity (series LLSTO1), confirming the importance of a composition rather than a crystal symmetry on conductivity.

Samples displaying higher Sr or La contents exhibit lower conductivity values. From these considerations, Li content has a greater effect than La content, because it increases the charge carrier concentration and the amount of “efficient” A vacancy. As the sample composition approaches the vacancy percolation threshold, conductivity values decrease by several orders of magnitude.

Finally, secondary phases were detected in samples with higher Sr or Li contents. In the first case, the SrTiO_3_ phase was formed in Sr-rich samples. In Li-rich samples, the formation of LiOH or Li_2_CO_3_ phases was favored by the H/Li exchange with adsorbed water.^20^ The carbonation of samples was favored in Li-rich samples, with compositions near La_1/2_Li_1/2_TiO_3_, but not in La- or Sr-rich samples. The sample carbonation reduces the “grain boundary” contribution to conductivity.

## Conclusions

The La_1/2_Li_1/2–2*x*_Sr_*x*_TiO_3_ (0 < *x* ≤ 0.25) (LLSTO3) series was investigated by XRD, NMR, and electrical conductivity techniques. In this series, the substitution of two Li by Sr generates nominal vacancies (□_A_) at A-sites of the perovskite, becoming progressively ordered with increasing Sr contents. The maximum conductivity, 4 × 10^–2^ S cm^–1^, is achieved when a small amount of Sr is added to the La_1/2_Li_1/2_TiO_3_ composition (*x* = 0).

The comparison of the solid solution LLSTO3 (Li_1/2–2*x*_Sr_*x*_La_1/2_TiO_3_), analyzed here, with other Sr-doped series, LLSTO1 (Li_1/2–*x*_Sr_2*x*_La_1/2–*x*_TiO_3_) and LLSTO2 (Li_*x*_Sr_*x*_La_2/3–*x*_TiO_3_), has shown that the so-called “effective” vacancy (*n*_t_), given by the ([Li] + □_A_) expression, is more adequate to describe conductivity results than nominal vacancy (□_A_).

The variation in dc-conductivity with Li and *n*_t_ contents showed that samples with high vacancy concentration (La- or Sr-rich) display 2D mobility, but those with low vacancy concentrations (Li-rich) display 3D mobility of lithium. In the analyzed series, the percolation of A-site vacancies considerably increases the Li mobility along the conduction channels, while the presence of La(Sr) ions blocks the conduction paths.

The Li mobility was discussed in terms of short and long distances. The short mobility of lithium is favored inside the unit cells, but the long-range mobility of lithium is easier when *n*_t_ is higher than the percolation threshold (*n*_p_). Li conductivity decreases dramatically when *n*_t_ approaches the percolation threshold of 2D and 3D systems. In conclusion, the comparison of the analyzed series reinforces the idea that *n*_t_ is the most relevant parameter for understanding ionic mobility in fast ion conductors with a perovskite structure.

## Supporting Information Available

Dependence of nominal and “effective” vacancies on the La content, structural model used for Rietveld refinement, and data obtained from the structural refinements (PDF)
