# Supplementary material for: Influence of Cation Vacancies on Li Conductivity of La1/2Li1/2–2xSrxTiO3 Perovskites (0 < x ≤ 0.25): The Role of Nominal and Effective Vacancies
Source: ACS Appl Energy Mater. 2023 Mar 2;6(5):2758–67. doi: 10.1021/acsaem.2c03519 (PMC10015983; doi:10.1021/acsaem.2c03519)
Supplement: Supplementary file 1 — ae2c03519_si_001.pdf [file ae2c03519_si_001.pdf]

Supporting Information

Influence of cation vacancies on Li conductivity of $La_{1/2}Li_{1/2-2x}Sr_xTiO_3$ perovskites (0 < x ≤0.25). The role of nominal and effective vacancies.

Wilmer Bucheli[1], Ricardo Jiménez[1], Jesús Sanz[1], Maria Eugenia Sotomayor[2] and Alejandro Varez[2]*

[1]Dpto. Energía. Instituto Ciencia de Materiales (ICMM-CSIC). 28049 Madrid. Spain.

[2]Dpto. de Ciencia e Ingeniería de Materiales e Ing. Química, IAAB, Universidad Carlos III de Madrid. Av. Universidad 30, 28911 Leganes. Spain.

*Corresponding Author: alejandro.varez@ing.uc3m.es

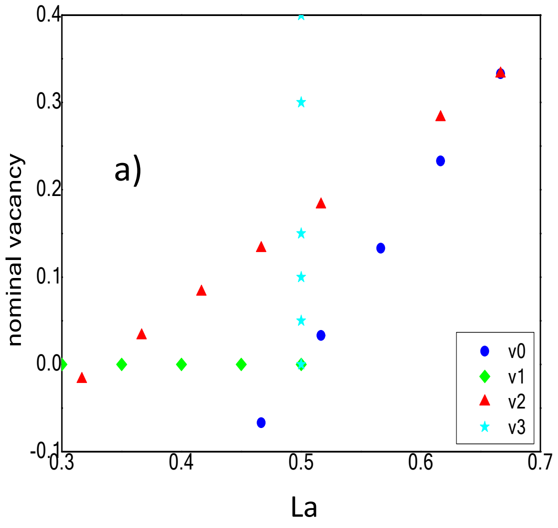

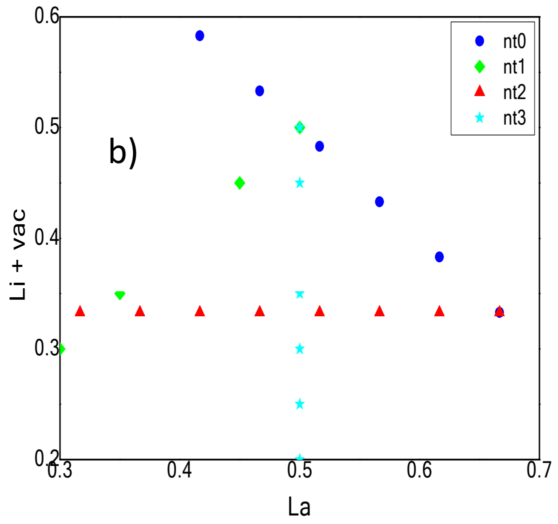

Figure SI-1.- Dependence of nominal (a) and "effective" (b) vacancy on the La content. In both figures, values deduced in previous works are included.

Table SI-1. - Positional parameters for tetragonal perovskites (S.G.: P4/mmm (Nº 123)) used in the structural refinement (Li has not been considered in the refinement).

S.G:P4/mmm(Nº 123)		
Atom	**Position**	***Atomic* coordinates**
La1, Sr1	1a	0,0,0
La2, Sr2	1b	0,0, ½
Ti	2h	½, ½ ,z
O1	4i	0, ½, z
O2	1c	½, ½, 0
O3	1d	½, ½, ½

Table SI-2. - Structural parameters deduced from X-Ray powder diffraction data of $La_{1/2}Li_{1/2-2x}Sr_xTiO_3$ perovskites. All the patterns were refined in the P4/mmm Space Group (N° 123) (see reference 4).

x	*0.1*	*0.15*	*0.19*	*0.20*	*0.225*	*0.250*
a (Å)	*3.8808(1)*	*3.8809(1)*	*3.8828(3)*	*3.8809(1)*	*3.8816(3)*	*3.8816(2)*
c(Å)	*7.7645(3)*	*7.7590(2)*	*7.766(1)*	*7.7607(5)*	*7.7598(9)*	*7.7658(6)*
Ti (z)	*0.2449(1)*	*0.2578(2)*	*0.2579(1)*	*0.2652(2)*	*0.2507(4)*	*0.2585(2)*
O1 (z)	*0.2474(2)*	*0.2516(2)*	*0.2434(1)*	*0.2361(3)*	*0.2119(8)*	*0.2409(2)*
$B_{La1(Sr)}$ *(Å^2)*	*0.850(2)*	*0.996(3)*	*0.973(2)*	*0.577(2)*	*0.962(6)*	*1.28(1)*
$B_{La2(Sr)}$ *(Å^2)*	*0.335(2)*	*0.383(3)*	*0.432(2)*	*0.347(4)*	*0.404(9)*	*1.42(1)*
B_{Ti} *(Å^2)*	*0.171(2)*	*0.414(2)*	*0.401(1)*	*0.326(2)*	*0.617(4)*	*1.32(1)*
B_{O1} *(Å^2)*	*0.31(5)*	*0.90(5)*	*1.01(8)*	*0.14(2)*	*0.77(7)*	*1.09(6)*
B_{O2} *(Å^2)*	*0.74(5)*	*0.98(5)*	*1.42(8)*	*0.92(4)*	*1.79(6)*	*2.62(5)*
B_{O3} *(Å^2)*	*1.66(3)*	*1.35(3)*	*1.08(4)*	*1.76(2)*	*1.14(3)*	*1.38(2)*
dTi-O1 (Å)	*1.9017(7)*	*2.0002(9)*	*2.0034(9)*	*2.058(2)*	*2.0908(9)*	*2.008(1)*
dTi-O2 (Å)	*1.9806(7)*	*1.8793(9)*	*1.8804(9)*	*1.822(2)*	*1.7891(9)*	*1.874(1)*
4 x dTi-O3 (Å)	*1.9405(1)*	*1.9411(1)*	*1.9447(1)*	*1.9535(3)*	*1.9414(2)*	*1.9458(2)*
4 x dLa1-O1 (Å)	*2.7441(1)*	*2.7443(1*	*2.7456(1)*	*2.7442(1)*	*2.7447(2)*	*2.7447(1)*
8 x dLa1-O3 (Å)	*2.7304(9)*	*2.7526(9)*	*2.7098(8)*	*2.6688(2)*	*2.7074(7)*	*2.6956(11)*
4 x dLa2-O2 (Å)	*2.7441(1)*	*2.7443(1)*	*2.7456(1)*	*2.7442(1)*	*2.7447(1)*	*2.7447(1)*
8 x dLa2-O3 (Å)	*2.7590(9)*	*2.7349(9)*	*2.7825(8)*	*2.8213(2)*	*2.7813(8)*	*2.7956(11)*
La1(occ)	*0.658*	*0.660(7)*	*0.716*	*0.690*	*0.763(1)*	*0.685(3)*
Sr1(occ)	*0.084*	*0.06(1)*	*0.037*	*0.072*	*0.066(2)*	*0.115(4)*
La2(occ)	*0.342*	*0.340(7)*	*0.284*	*0.310*	*0.237(1)*	*0.315(3)*
Sr2(occ)	*0.116*	*0.24(1)*	*0.343*	*0.328*	*0.384(2)*	*0.385(4)*
R_p	*11.7*	*9.85*	*10.2*	*11.4*	*11.5*	*10.2*
R_{wp}	*17.7*	*13.0*	*13.7*	*15.0*	*15.5*	*13.6*
χ^2	*4.62*	*2.69*	*3.41*	*3.11*	*4.75*	*3.75*
R_B	*11.1*	*14.0*	*9.42*	*9.04*	*8.75*	*9.44*
R_F	*16.3*	*9.68*	*9.10*	*9.25*	*7.19*	*11.3*
